# Crystal structure of (*E*)-1-{[(3,5-di­methyl­phen­yl)imino]­meth­yl}naphthalen-2-ol

**DOI:** 10.1107/S2056989015011548

**Published:** 2015-06-20

**Authors:** Ahmed M. Abu-Dief, Mohammed S. M. Abdelbaky, Santiago Garcia-Granda

**Affiliations:** a Department of Chemistry, Faculty of Science, Sohag University, 82524 Sohag, Egypt; b Department of Physical and Analytical Chemistry, Faculty of Chemistry, Oviedo University-CINN, Oviedo 33006, Spain

**Keywords:** crystal structure, Schiff base, naphthalen-2-ol, imino, hydrogen bonding

## Abstract

The title compound, C_19_H_17_NO, has an *E* conformation about the N=C bond. The mol­ecule is relatively planar, with the benzene ring and naphthalene ring plane being inclined to one another by 4.28 (10)°. There is an intra­molecular O—H⋯N hydrogen bond generating an *S*(6) ring motif. In the crystal, mol­ecules are linked *via* C—H⋯O hydrogen bonds, forming chains propagating along [100]. Within the chains there are π–π inter­actions involving the benzene ring and the naphthalene ring system of an adjacent mol­ecule [inter-centroid distance = 3.6405 (14) Å].

## Related literature   

For the diverse applications and biological activities of Schiff bases, see: Schiff (1864 ▸); Dutta & Das (1988 ▸); Chandra & Sangeetika (2004 ▸); Cozzi (2004 ▸). For the biological activity and optical properties of Schiff bases derived from 2-hy­droxy­napthaldehyde, see: Abdel-Rahman *et al.* (2013*a* ▸,*b* ▸, 2014 ▸); Abu-Dief *et al.* (2013 ▸).
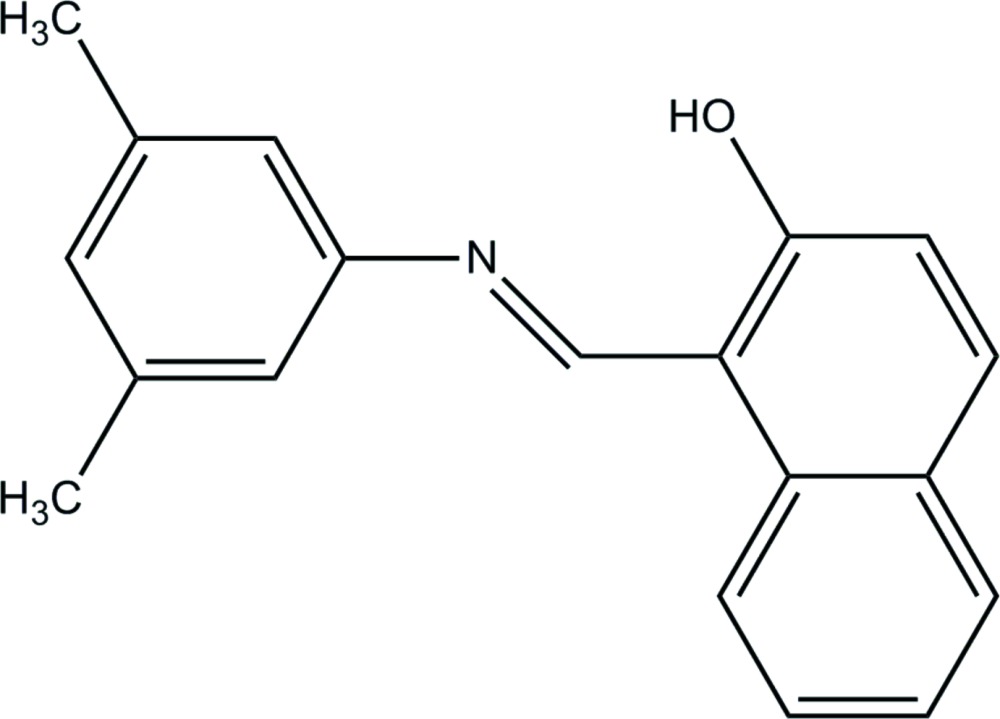


## Experimental   

### Crystal data  

C_19_H_17_NO
*M* *_r_* = 275.33
Orthorhombic, 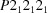

*a* = 6.2463 (2) Å
*b* = 10.2438 (3) Å
*c* = 23.0533 (8) Å
*V* = 1475.08 (8) Å^3^
*Z* = 4
Cu *K*α radiation
μ = 0.60 mm^−1^
*T* = 293 K
0.73 × 0.12 × 0.09 mm

### Data collection  

Oxford Diffraction Xcalibur (Ruby, Gemini) diffractometer
Absorption correction: analytical (*CrysAlis PRO*; Oxford Diffraction, 2010 ▸) *T*_min_ = 0.915, *T*_max_ = 0.94
8103 measured reflections
2834 independent reflections
1422 reflections with *I* > 2σ(*I*)
*R*_int_ = 0.032

### Refinement  

*R*[*F* ^2^ > 2σ(*F* ^2^)] = 0.041
*wR*(*F* ^2^) = 0.121
*S* = 1.09
1649 reflections
193 parameters
H-atom parameters constrained
Δρ_max_ = 0.17 e Å^−3^
Δρ_min_ = −0.14 e Å^−3^

### 

Data collection: *CrysAlis CCD* (Oxford Diffraction, 2010 ▸); cell refinement: *CrysAlis CCD*; data reduction: *CrysAlis RED* (Oxford Diffraction, 2010 ▸); program(s) used to solve structure: *SIR2011* (Burla *et al.*, 2015 ▸); program(s) used to refine structure: *SHELXL2014* (Sheldrick, 2015 ▸); molecular graphics: *Mercury* (Macrae *et al.*, 2008 ▸); software used to prepare material for publication: *SHELXL2014* and *PLATON* (Spek, 2009 ▸).

## Supplementary Material

Crystal structure: contains datablock(s) global, I. DOI: 10.1107/S2056989015011548/su5133sup1.cif

Structure factors: contains datablock(s) I. DOI: 10.1107/S2056989015011548/su5133Isup2.hkl

Supporting information file. DOI: 10.1107/S2056989015011548/su5133Isup3.cml

**Fig. 1.**
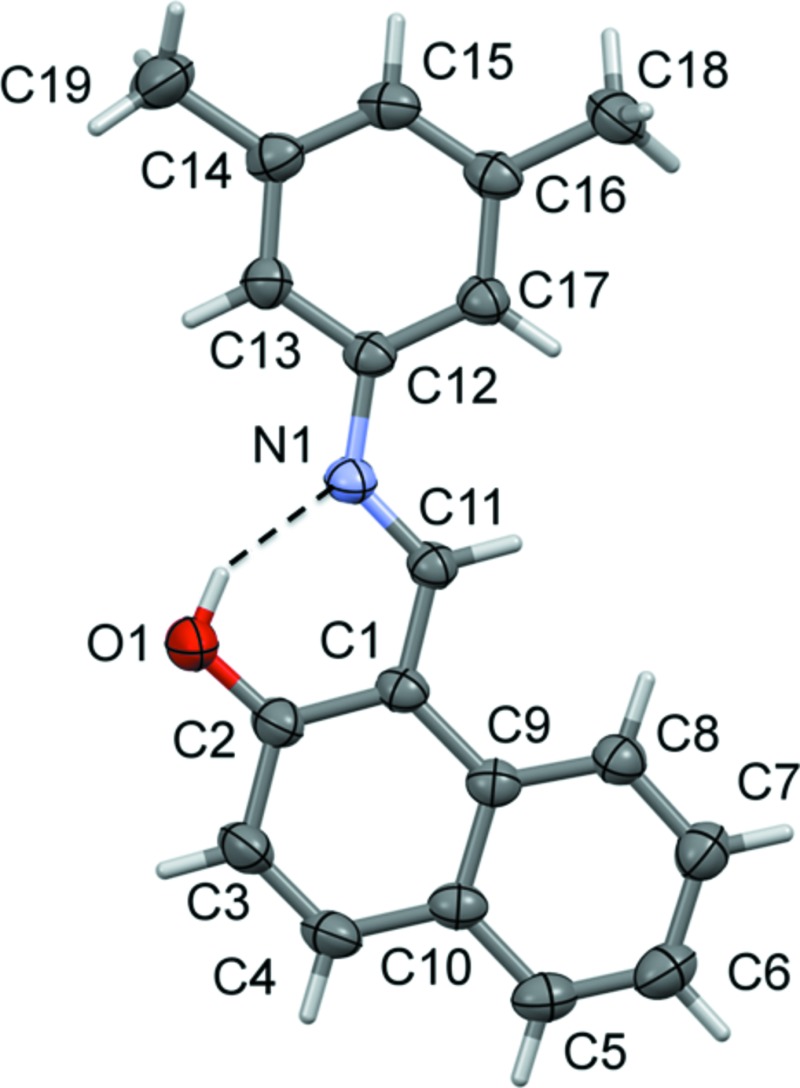
A view of the molecular structure of the title compound, with atom labelling. Displacement ellipsoids are drawn at the 50% probability level. The intramolecular O—H···N hydrogen bond is shown as a dashed line (see Table 1 for details).

**Fig. 2.**
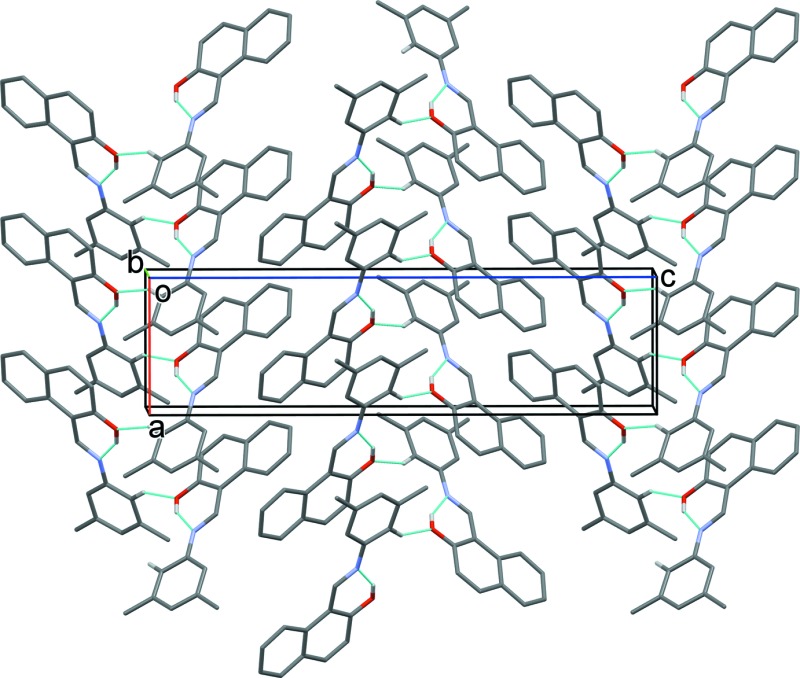
A view along the b axis of the crystal packing of the title compound. Hydrogen bonds are shown as dashed lines (see Table 1 for details). H atoms not involved in these interactions have been omitted for clarity.

. DOI: 10.1107/S2056989015011548/su5133fig1.tif
A view of the mol­ecular structure of the title compound, with atom labelling. Displacement ellipsoids are drawn at the 50% probability level. The intra­molecular O—H⋯N hydrogen bond is shown as a dashed line (see Table 1 for details).

b . DOI: 10.1107/S2056989015011548/su5133fig2.tif
A view along the *b* axis of the crystal packing of the title compound. Hydrogen bonds are shown as dashed lines (see Table 1 for details). H atoms not involved in these inter­actions have been omitted for clarity.

CCDC reference: 1406684

Additional supporting information:  crystallographic information; 3D view; checkCIF report

## Figures and Tables

**Table 1:** Hydrogen-bond geometry (, )

*D*H*A*	*D*H	H*A*	*D* *A*	*D*H*A*
O1H1N1	0.82	1.78	2.523(3)	149
C13H13O1^i^	0.93	2.62	3.492(3)	156

Symmetry code: (i) 
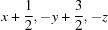
.

## supplementary crystallographic information

### S1. Structural commentary

Schiff bases, known as anils, imines or azomethines, have recently received considerable attention due to their good performance in coordination chemistry, unique anti-bacterial, anti-cancer, and herbicidal applications (Schiff, 1864; Abdel-Rahman *et al.*, 2013a,b,2014; Dutta & Das,1988). Studies showed that the presence of a lone pair of electrons in an sp^2^ hybridized orbital of the nitro­gen atom of the azomethine group is of considerable chemical and biological importance (Chandra & Sangeetika, 2004; Cozzi, 2004). In continuation of our inter­est in the chemical, herbicidal and biological properties of Schiff bases we synthesized the title compound as a potential anti-bacterial agent.

The title compound, has an *E* conformation about the N1═C11 bond, as illustrated in Fig. 1. The molecule is relatively planar with the benzene ring (C12—C17) and the naphthalene plane (C1—C10) being inclined to one another by 4.31 (10) °. There is an intra­molecular O—H···N hydrogen bond generating an S(6) ring motif (Table 1 and Fig. 1).

In the crystal, molecules are linked *via* C—H···O hydrogen bonds (Table 1) forming chains propagating along [100], as shown in Fig. 2. Within the chains there are π-π inter­actions involving the naphthalene ring system and the benzene ring of an adjacent molecule [Cg1···Cg3^i^ = 3.6405 (14) Å; Cg1 and Cg3 are the centroids of rings C1—C4/C9/C10 and C12—C17; symmetry code: (i) x - 1, y, z].

### S2. Synthesis and crystallization

The title compound was prepared by treating 3,5-di­methyl­aniline (0.38 ml, 3 mmol) in 30 ml of dry ethanol with 2-hy­droxy­napthaldehyde (0.52 g, 3 mmol) with vigorous stirring at 343 K for 2 h. The reaction mixture was then left to stand at room temperature for 30 min. The yellow crystals were collected and washed several times in ethanol.

### S3. Refinement details

Crystal data, data collection and structure refinement details are summarized in Table 2. All the H atoms were positioned geometrically and refined using a riding model: O—H = 0.82 Å, C—H = 0.93 - 9.96 Å with U_iso_(H) = 1.5U_eq_(O,C) for hydroxyl and methyl H atoms and 1.2U_eq_(C) for other H atoms. In the final cycles of refinement, in the absence of significant anomalous scattering effects, 1185 Friedel pairs were merged and Δf ' set to zero.

### Crystal data

**Table:**

C_19_H_17_NO	*D*_x_ = 1.240 Mg m^−^^3^
*M**_r_* = 275.33	Cu *K*α radiation, λ = 1.54184 Å
Orthorhombic, *P*2_1_2_1_2_1_	Cell parameters from 2417 reflections
*a* = 6.2463 (2) Å	θ = 3.8–69.9°
*b* = 10.2438 (3) Å	µ = 0.60 mm^−^^1^
*c* = 23.0533 (8) Å	*T* = 293 K
*V* = 1475.08 (8) Å^3^	Prism, colourless
*Z* = 4	0.73 × 0.12 × 0.09 mm
*F*(000) = 584	

### Data collection

**Table:**

Oxford Diffraction Xcalibur (Ruby, Gemini) diffractometer	2834 independent reflections
Radiation source: Enhance (Cu) X-ray Source	1422 reflections with *I* > 2σ(*I*)
Graphite monochromator	*R*_int_ = 0.032
Detector resolution: 10.2673 pixels mm^-1^	θ_max_ = 70.4°, θ_min_ = 3.8°
ω scans	*h* = −7→6
Absorption correction: analytical (*CrysAlis PRO*; Oxford Diffraction, 2010)	*k* = −12→12
*T*_min_ = 0.915, *T*_max_ = 0.94	*l* = −27→28
8103 measured reflections	

### Refinement

**Table:**

Refinement on *F*^2^	0 restraints
Least-squares matrix: full	Hydrogen site location: inferred from neighbouring sites
*R*[*F*^2^ > 2σ(*F*^2^)] = 0.041	H-atom parameters constrained
*wR*(*F*^2^) = 0.121	*w* = 1/[σ^2^(*F*_o_^2^) + (0.0778*P*)^2^ + 0.0057*P*] where *P* = (*F*_o_^2^ + 2*F*_c_^2^)/3
*S* = 1.09	(Δ/σ)_max_ = 0.001
1649 reflections	Δρ_max_ = 0.17 e Å^−^^3^
193 parameters	Δρ_min_ = −0.14 e Å^−^^3^

### Special details

**Table:**

Geometry. All esds (except the esd in the dihedral angle between two l.s. planes) are estimated using the full covariance matrix. The cell esds are taken into account individually in the estimation of esds in distances, angles and torsion angles; correlations between esds in cell parameters are only used when they are defined by crystal symmetry. An approximate (isotropic) treatment of cell esds is used for estimating esds involving l.s. planes.

### Fractional atomic coordinates and isotropic or equivalent isotropic displacement parameters (Å^2^)

**Table:**

	*x*	*y*	*z*	*U*_iso_*/*U*_eq_	
O1	0.6500 (4)	0.7247 (2)	0.05918 (10)	0.0874 (7)	
H1	0.7473	0.6710	0.0584	0.131*	
N1	0.8802 (3)	0.53539 (18)	0.09101 (9)	0.0551 (5)	
C1	0.5709 (3)	0.6003 (2)	0.14386 (10)	0.0511 (5)	
C2	0.5267 (4)	0.7006 (2)	0.10264 (13)	0.0641 (7)	
C3	0.3348 (5)	0.7757 (2)	0.10869 (14)	0.0740 (8)	
H3	0.3038	0.8404	0.0817	0.089*	
C4	0.1983 (4)	0.7542 (3)	0.15291 (13)	0.0703 (7)	
H4	0.0740	0.8040	0.1554	0.084*	
C5	0.0928 (5)	0.6360 (3)	0.24126 (13)	0.0712 (7)	
H5	−0.0293	0.6877	0.2437	0.085*	
C6	0.1263 (5)	0.5409 (3)	0.28162 (13)	0.0794 (8)	
H6	0.0271	0.5275	0.3111	0.095*	
C7	0.3088 (5)	0.4643 (3)	0.27857 (12)	0.0728 (7)	
H7	0.3322	0.3997	0.3062	0.087*	
C8	0.4552 (4)	0.4833 (3)	0.23502 (11)	0.0614 (6)	
H8	0.5772	0.4314	0.2338	0.074*	
C9	0.4256 (4)	0.5796 (2)	0.19192 (10)	0.0524 (5)	
C10	0.2386 (4)	0.6573 (2)	0.19625 (11)	0.0577 (6)	
C11	0.7513 (4)	0.5198 (2)	0.13492 (10)	0.0508 (5)	
H11	0.7791	0.4533	0.1613	0.061*	
C12	1.0636 (4)	0.4608 (2)	0.07758 (10)	0.0516 (5)	
C13	1.1710 (4)	0.4957 (2)	0.02723 (11)	0.0590 (6)	
H13	1.1194	0.5640	0.0046	0.071*	
C14	1.3542 (4)	0.4297 (2)	0.01026 (12)	0.0639 (6)	
C15	1.4288 (4)	0.3287 (2)	0.04512 (12)	0.0644 (7)	
H15	1.5511	0.2834	0.0340	0.077*	
C16	1.3257 (4)	0.2940 (2)	0.09578 (12)	0.0599 (6)	
C17	1.1405 (4)	0.3596 (2)	0.11176 (11)	0.0562 (6)	
H17	1.0680	0.3359	0.1454	0.067*	
C18	1.4130 (5)	0.1868 (3)	0.13355 (14)	0.0790 (8)	
H18A	1.5003	0.1295	0.1106	0.119*	
H18B	1.2965	0.1383	0.1501	0.119*	
H18C	1.4979	0.2241	0.1640	0.119*	
C19	1.4706 (6)	0.4694 (3)	−0.04430 (15)	0.0950 (11)	
H19A	1.4351	0.5581	−0.0538	0.142*	
H19B	1.4286	0.4132	−0.0756	0.142*	
H19C	1.6222	0.4622	−0.0382	0.142*	

### Atomic displacement parameters (Å^2^)

**Table:**

	*U*^11^	*U*^22^	*U*^33^	*U*^12^	*U*^13^	*U*^23^
O1	0.0695 (12)	0.0857 (13)	0.1071 (15)	0.0178 (11)	0.0232 (13)	0.0409 (12)
N1	0.0420 (9)	0.0567 (9)	0.0667 (11)	0.0027 (9)	0.0022 (10)	0.0052 (8)
C1	0.0410 (10)	0.0505 (10)	0.0618 (13)	−0.0015 (9)	−0.0034 (11)	−0.0022 (9)
C2	0.0518 (13)	0.0585 (12)	0.0819 (17)	0.0050 (12)	0.0038 (14)	0.0120 (12)
C3	0.0656 (15)	0.0588 (13)	0.098 (2)	0.0161 (14)	0.0032 (18)	0.0149 (13)
C4	0.0542 (13)	0.0616 (13)	0.095 (2)	0.0151 (12)	0.0018 (15)	−0.0004 (13)
C5	0.0516 (13)	0.0851 (17)	0.0770 (17)	0.0037 (13)	0.0034 (14)	−0.0138 (14)
C6	0.0640 (16)	0.106 (2)	0.0685 (17)	−0.0055 (18)	0.0119 (15)	−0.0069 (16)
C7	0.0659 (16)	0.0889 (18)	0.0638 (15)	−0.0074 (16)	0.0016 (14)	0.0051 (13)
C8	0.0529 (13)	0.0698 (13)	0.0616 (14)	0.0036 (12)	−0.0019 (12)	0.0015 (11)
C9	0.0438 (11)	0.0546 (11)	0.0587 (13)	−0.0040 (10)	−0.0046 (11)	−0.0066 (10)
C10	0.0457 (11)	0.0606 (12)	0.0668 (15)	0.0009 (11)	−0.0022 (12)	−0.0110 (11)
C11	0.0428 (10)	0.0513 (10)	0.0583 (13)	−0.0011 (9)	−0.0034 (10)	0.0025 (10)
C12	0.0405 (10)	0.0494 (10)	0.0648 (14)	−0.0027 (10)	−0.0009 (10)	−0.0009 (9)
C13	0.0590 (14)	0.0536 (10)	0.0645 (13)	0.0000 (11)	0.0065 (13)	0.0035 (10)
C14	0.0592 (14)	0.0570 (12)	0.0757 (16)	−0.0041 (12)	0.0136 (14)	−0.0058 (11)
C15	0.0512 (13)	0.0542 (11)	0.0877 (18)	0.0019 (11)	0.0070 (14)	−0.0090 (12)
C16	0.0475 (12)	0.0523 (11)	0.0801 (16)	−0.0003 (11)	−0.0040 (14)	−0.0031 (11)
C17	0.0474 (11)	0.0554 (11)	0.0658 (14)	−0.0006 (11)	−0.0007 (12)	0.0042 (10)
C18	0.0670 (16)	0.0714 (15)	0.099 (2)	0.0149 (14)	−0.0076 (17)	0.0115 (14)
C19	0.096 (2)	0.0869 (18)	0.102 (2)	0.0041 (19)	0.046 (2)	0.0044 (17)

### Geometric parameters (Å, º)

**Table:**

O1—C2	1.287 (3)	C8—H8	0.9300
O1—H1	0.8200	C9—C10	1.417 (3)
N1—C11	1.304 (3)	C11—H11	0.9300
N1—C12	1.411 (3)	C12—C13	1.387 (3)
C1—C11	1.411 (3)	C12—C17	1.388 (3)
C1—C2	1.427 (3)	C13—C14	1.385 (3)
C1—C9	1.448 (3)	C13—H13	0.9300
C2—C3	1.431 (4)	C14—C15	1.391 (4)
C3—C4	1.347 (4)	C14—C19	1.509 (4)
C3—H3	0.9300	C15—C16	1.380 (4)
C4—C10	1.431 (4)	C15—H15	0.9300
C4—H4	0.9300	C16—C17	1.388 (3)
C5—C6	1.363 (4)	C16—C18	1.504 (3)
C5—C10	1.398 (4)	C17—H17	0.9300
C5—H5	0.9300	C18—H18A	0.9600
C6—C7	1.386 (4)	C18—H18B	0.9600
C6—H6	0.9300	C18—H18C	0.9600
C7—C8	1.372 (4)	C19—H19A	0.9600
C7—H7	0.9300	C19—H19B	0.9600
C8—C9	1.412 (3)	C19—H19C	0.9600
			
C2—O1—H1	109.5	N1—C11—H11	118.8
C11—N1—C12	127.3 (2)	C1—C11—H11	118.8
C11—C1—C2	118.6 (2)	C13—C12—C17	120.0 (2)
C11—C1—C9	121.8 (2)	C13—C12—N1	115.9 (2)
C2—C1—C9	119.6 (2)	C17—C12—N1	124.1 (2)
O1—C2—C1	122.7 (2)	C14—C13—C12	120.7 (2)
O1—C2—C3	118.3 (2)	C14—C13—H13	119.7
C1—C2—C3	119.0 (2)	C12—C13—H13	119.7
C4—C3—C2	121.1 (2)	C13—C14—C15	118.5 (2)
C4—C3—H3	119.5	C13—C14—C19	120.1 (3)
C2—C3—H3	119.5	C15—C14—C19	121.4 (2)
C3—C4—C10	122.0 (2)	C16—C15—C14	121.6 (2)
C3—C4—H4	119.0	C16—C15—H15	119.2
C10—C4—H4	119.0	C14—C15—H15	119.2
C6—C5—C10	121.2 (3)	C15—C16—C17	119.2 (2)
C6—C5—H5	119.4	C15—C16—C18	120.6 (2)
C10—C5—H5	119.4	C17—C16—C18	120.2 (3)
C5—C6—C7	119.8 (3)	C16—C17—C12	120.0 (2)
C5—C6—H6	120.1	C16—C17—H17	120.0
C7—C6—H6	120.1	C12—C17—H17	120.0
C8—C7—C6	120.3 (3)	C16—C18—H18A	109.5
C8—C7—H7	119.8	C16—C18—H18B	109.5
C6—C7—H7	119.8	H18A—C18—H18B	109.5
C7—C8—C9	121.8 (2)	C16—C18—H18C	109.5
C7—C8—H8	119.1	H18A—C18—H18C	109.5
C9—C8—H8	119.1	H18B—C18—H18C	109.5
C8—C9—C10	116.8 (2)	C14—C19—H19A	109.5
C8—C9—C1	124.0 (2)	C14—C19—H19B	109.5
C10—C9—C1	119.2 (2)	H19A—C19—H19B	109.5
C5—C10—C9	120.1 (2)	C14—C19—H19C	109.5
C5—C10—C4	120.8 (2)	H19A—C19—H19C	109.5
C9—C10—C4	119.1 (2)	H19B—C19—H19C	109.5
N1—C11—C1	122.4 (2)		
			
C11—C1—C2—O1	−3.3 (4)	C1—C9—C10—C4	0.0 (3)
C9—C1—C2—O1	179.5 (2)	C3—C4—C10—C5	−179.2 (3)
C11—C1—C2—C3	174.9 (2)	C3—C4—C10—C9	−1.3 (4)
C9—C1—C2—C3	−2.2 (4)	C12—N1—C11—C1	−179.6 (2)
O1—C2—C3—C4	179.3 (3)	C2—C1—C11—N1	1.2 (3)
C1—C2—C3—C4	1.0 (4)	C9—C1—C11—N1	178.2 (2)
C2—C3—C4—C10	0.8 (4)	C11—N1—C12—C13	178.5 (2)
C10—C5—C6—C7	0.6 (4)	C11—N1—C12—C17	−2.8 (4)
C5—C6—C7—C8	−0.3 (4)	C17—C12—C13—C14	0.6 (4)
C6—C7—C8—C9	−0.5 (4)	N1—C12—C13—C14	179.4 (2)
C7—C8—C9—C10	0.9 (4)	C12—C13—C14—C15	−0.6 (4)
C7—C8—C9—C1	−177.6 (2)	C12—C13—C14—C19	−179.4 (3)
C11—C1—C9—C8	3.1 (3)	C13—C14—C15—C16	−0.4 (4)
C2—C1—C9—C8	−179.8 (2)	C19—C14—C15—C16	178.4 (3)
C11—C1—C9—C10	−175.3 (2)	C14—C15—C16—C17	1.4 (4)
C2—C1—C9—C10	1.7 (3)	C14—C15—C16—C18	−178.3 (3)
C6—C5—C10—C9	−0.1 (4)	C15—C16—C17—C12	−1.4 (3)
C6—C5—C10—C4	177.8 (3)	C18—C16—C17—C12	178.3 (2)
C8—C9—C10—C5	−0.6 (3)	C13—C12—C17—C16	0.4 (3)
C1—C9—C10—C5	177.9 (2)	N1—C12—C17—C16	−178.3 (2)
C8—C9—C10—C4	−178.5 (2)		

### Hydrogen-bond geometry (Å, º)

**Table:**

*D*—H···*A*	*D*—H	H···*A*	*D*···*A*	*D*—H···*A*
O1—H1···N1	0.82	1.78	2.523 (3)	149
C13—H13···O1^i^	0.93	2.62	3.492 (3)	156

Symmetry code: (i) *x*+1/2, −*y*+3/2, −*z*.

## References

[bb1] Abdel-Rahman, L. H., El-Khatib, R. M., Nassr, L. A. E. & Abu-Dief, A. M. (2013*a*). *J. Mol. Struct.* **1040**, 9–18.

[bb2] Abdel-Rahman, L. H., El-Khatib, R. M., Nassr, L. A. E., Abu-Dief, A. M., Ismael, M. & Seleem, A. A. (2014). *Spectrochim. Acta*, **117**, 366–378. 10.1016/j.saa.2013.07.056 24001978

[bb3] Abdel-Rahman, L. H., El-Khatib, R. M., Nassr, L. A. E., Abu-Dief, A. M. & Lashin, F. E. (2013*b*). *Spectrochim. Acta*, **111**, 266–276. 10.1016/j.saa.2013.03.061 23665616

[bb4] Abu-Dief, A. M., Díaz-Torres, R., Sañudo, E. C., Abdel-Rahman, L. H. & Aliaga-Alcalde, N. (2013). *Polyhedron*, **64**, 203–208.

[bb5] Burla, M. C., Caliandro, R., Carrozzini, B., Cascarano, G. L., Cuocci, C., Giacovazzo, C., Mallamo, M., Mazzone, A. & Polidori, G. (2015). *J. Appl. Cryst.* **48**, 306–309.

[bb6] Chandra, S. & Sangeetika, J. (2004). *J. Indian Chem. Soc.* **81**, 203–206.

[bb7] Cozzi, P. G. (2004). *Chem. Soc. Rev.* **33**, 410–421.

[bb8] Dutta, R. L. & Das, B. R. (1988). *J. Sci. Ind. Res.* **7**, 547–555.

[bb9] Macrae, C. F., Bruno, I. J., Chisholm, J. A., Edgington, P. R., McCabe, P., Pidcock, E., Rodriguez-Monge, L., Taylor, R., van de Streek, J. & Wood, P. A. (2008). *J. Appl. Cryst.* **41**, 466–470.

[bb10] Oxford Diffraction (2010). *CrysAlis PRO*, *CrysAlis CCD* and *CrysAlis RED*. Oxford Diffraction Ltd, Yarnton, England.

[bb11] Schiff, H. (1864). *Chem. Pharm. Suppl.* **3**, 343.

[bb12] Sheldrick, G. M. (2015). *Acta Cryst.* C**71**, 3–8.

[bb13] Spek, A. L. (2009). *Acta Cryst.* D**65**, 148–155. 10.1107/S090744490804362X PMC2631630 19171970

