# Supplementary figures and images for: Crystal structure of (E)-1-{[(3,5-di­methyl­phen­yl)imino]­meth­yl}naphthalen-2-ol
Source: Acta Crystallogr E Crystallogr Commun. 2015 Jun 20;71(Pt 7):o496–7. doi: 10.1107/S2056989015011548 (PMC4518976; doi:10.1107/S2056989015011548)

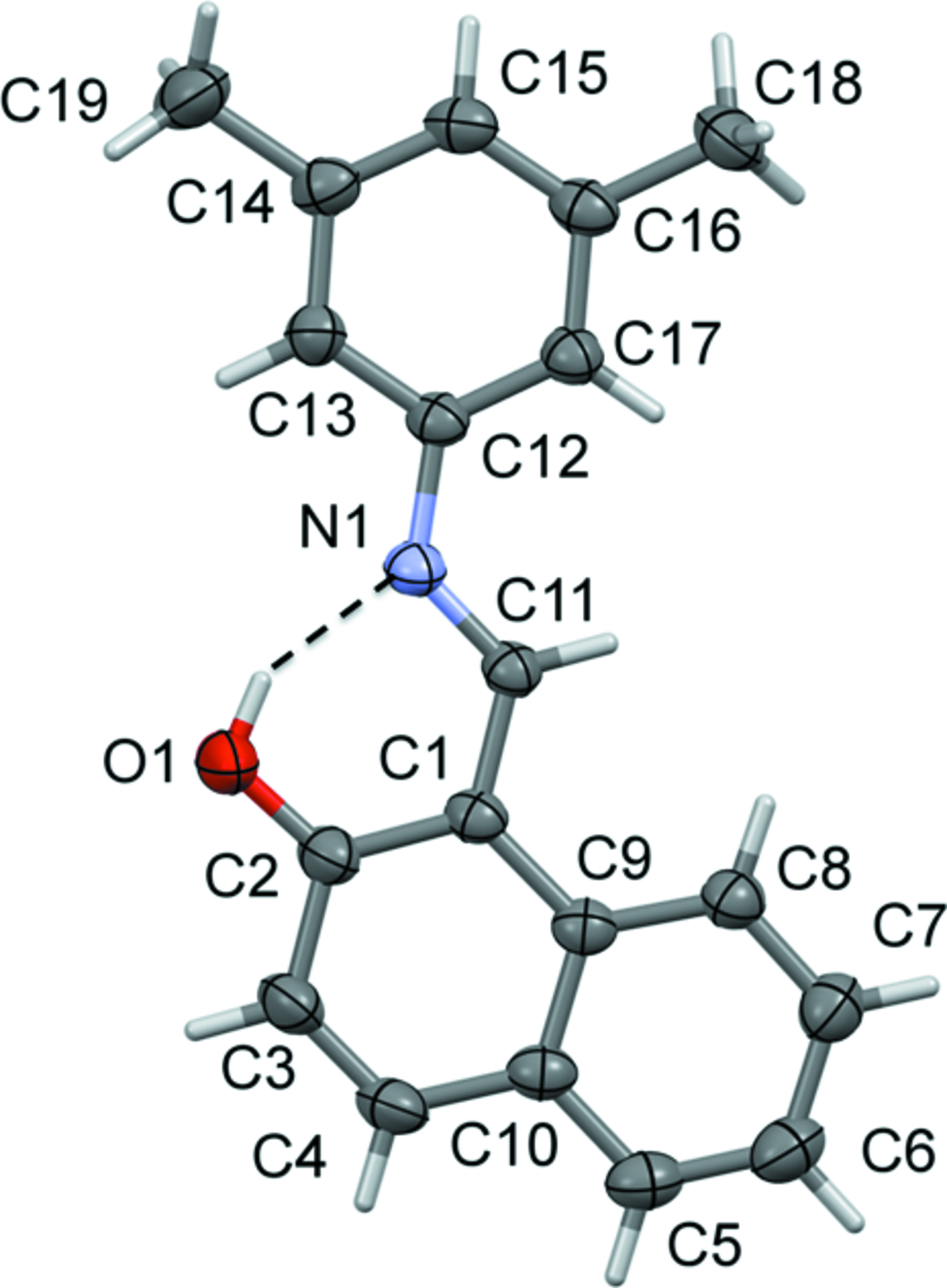

Supplement: Supplementary file 4 [file e-71-0o496-fig1.tif]

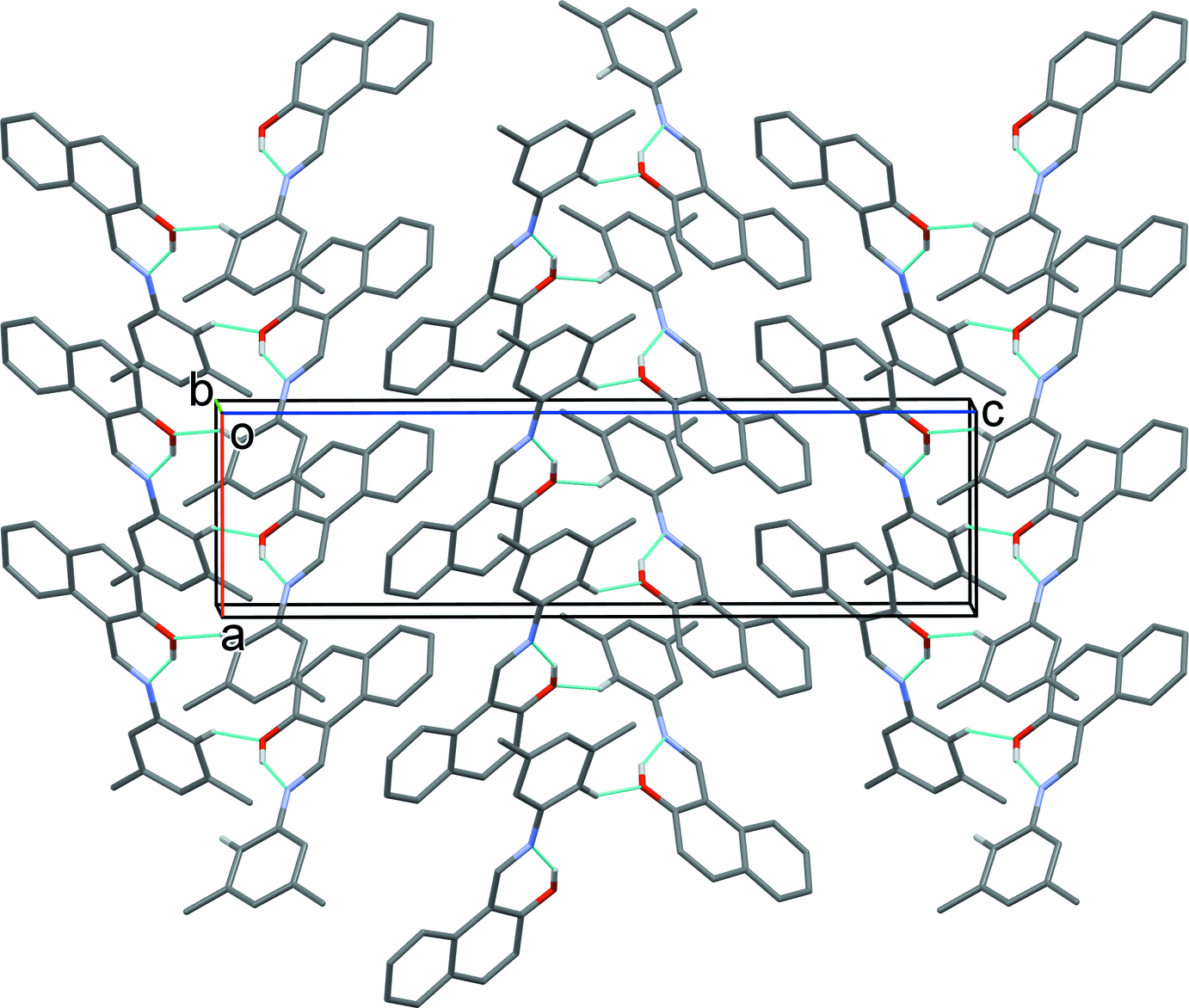

Supplement: Supplementary file 5 [file e-71-0o496-fig2.tif]
